# Draft Genome Sequences of *Pseudomonas* sp. Isolates Recovered from Ghanaian Fish Food Samples in 2018

**DOI:** 10.1128/MRA.01332-20

**Published:** 2021-02-11

**Authors:** Laura Wessels, Felix Reich, Silvia Schmoger, Johannes Pucher, Amy Atter, Annemarie Käsbohrer, Jens Andre Hammerl

**Affiliations:** a German Federal Institute for Risk Assessment, Biological Safety Department, Food Hygiene and Technology, Supply Chain, Food Defense Unit, Berlin, Germany; b German Federal Institute for Risk Assessment, Biological Safety Department, Epidemiology, Zoonosis and Antimicrobial Resistance Unit, Berlin, Germany; c German Federal Institute for Risk Assessment, Experimental Toxicology and ZEBET Department, Animal Husbandry, Aquaculture and Reference Material Unit, Berlin, Germany; d Council for Scientific and Industrial Research, Food Research Institute, Microbiology and Mushroom Research Division, Accra, Ghana; e University of Veterinary Medicine, Veterinary Public Health and Epidemiology Unit, Vienna, Austria; University of Arizona

## Abstract

The genus *Pseudomonas* represents a broad diversity of opportunistic and pathogenic species that are able to colonize a wide range of ecological niches. Here, we report on draft genome sequences of 35 *Pseudomonas* sp. isolates that were recovered from small processed Ghanaian fishes offered at food markets in 2018.

## ANNOUNCEMENT

*Pseudomonadaceae* are Gram-negative bacteria, of which some species are associated with animal, plant, and human diseases (1). Besides broad intrinsic resistance to different beta-lactams (2), many *Pseudomonas* species also produce exopolysaccharides involved in the formation of biofilms (3). These traits make them hard to treat, i.e., in food production, where they are involved in food spoilage (4). While detailed information on the diversity of *Pseudomonadaceae* exists (5), genomic data for food-associated isolates from middle income countries are rare.

Within the LEAP AGRI program-funded project SmallFishFood (https://smallfishfood.org), 104 samples of processed small fish were taken from five Ghanaian markets in November 2018 to assess the food safety and nutritional quality. For microbiological investigation, individual samples were pooled into batches for each fish species and market, prepared, and subjected to cultivation as previously described (6). *Pseudomonas* isolates were recovered from Brilliance Escherichia coli/coliform agar (Oxoid, Wesel, Germany) after incubation at 37°C for 20 to 24 h. Species confirmation was conducted using the direct transfer method on a matrix-assisted laser desorption ionization–time of flight (MALDI-TOF) Biotyper (Bruker Daltonik, Bremen, Germany) (7). Information on isolates, sources, and sampled markets is summarized in Table 1.

**TABLE 1:** Features of the *Pseudomonas* sp. isolates investigated in this study

				Raw sequencing results^*b*^				Assembly results				Annotation results			Accession no. for:	
Isolate	Yr of isolation	Sample source^*a*^	Market	Mean length (bp)	Total reads (M)	Total bases (M)	GC content (%)	No. of contigs	Genome size (bp)	*N*_50_ contig size (bp)	WGS-based species	Total no. of genes	Total no. of RNA genes	Total no. of pseudogenes	SRA	GenBank
20-MO00609	2018	WA pygmy herring	Accra	146	4.800810	704.385343	66.54	91	6,279,602	212,748	*P. aeruginosa*	5,874	65	72	SRS7648288	JADLJB000000000
20-MO00610	2018	WA pygmy herring	Techiman	146	4.475634	656.108908	66.55	110	6,251,217	258,224	*P. aeruginosa*	5,827	66	44	SRS7648292	JADLJC000000000
20-MO00611	2018	WA pygmy herring	Techiman	146	4.226370	619.125989	66.58	106	6,250,467	281,521	*P. aeruginosa*	5,826	66	40	SRS7648300	JADLJD000000000
20-MO00612	2018	Round sardinella	Accra	146	3.396300	498.454028	61.79	80	4,576,278	161,651	*P. fulva*	4,149	78	34	SRS7648311	JADLJE000000000
20-MO00613	2018	Round sardinella	Accra	146	3.187654	468.509732	61.71	78	4,577,142	170,695	*P. fulva*	4,153	78	34	SRS7648315	JADLJF000000000
20-MO00614	2018	African moonfish	Techiman	146	3.245274	475.860409	61.78	63	4,595,530	197,085	*P. fulva*	4,166	77	38	SRS7648316	JADLJG000000000
20-MO00615	2018	Bigeye grunt	Kumasi	146	2.604412	381.622970	61.75	85	4,927,608	331,604	*P. fulva*	4,493	82	61	SRS7648317	JADLJH000000000
20-MO00616	2018	African moonfish	Techiman	146	2.201970	322.159916	61.64	105	4,629,253	166,757	*P. fulva*	4,276	77	40	SRS7648318	JADLJI000000000
20-MO00617	2018	African moonfish	Techiman	146	3.256114	476.576303	61.85	71	4,596,202	193,568	*P. fulva*	4,172	77	41	SRS7648319	JADLJJ000000000
20-MO00618	2018	Bigeye grunt	Kumasi	146	2.618926	383.998382	61.77	86	4,926,499	244,295	*P. fulva*	4,496	81	61	SRS7648320	JADLJK000000000
20-MO00619	2018	Bigeye grunt	Accra	146	3.702502	543.679923	63.54	118	5,065,018	119,512	*P. guariconensis*	4,606	73	53	SRS7648289	JADLJL000000000
20-MO00620	2018	WA pygmy herring	Accra	146	3.643072	533.979303	62.60	70	5,419,544	226,217	*P. guariconensis*	4,935	74	39	SRS7648293	JADLJM000000000
20-MO00621	2018	Anchovy	Kumasi	146	3.901822	571.665460	63.31	162	5,288,586	92,968	*P. guariconensis*	4,706	74	47	SRS7648290	JADLJN000000000
20-MO00622	2018	Anchovy	Kumasi	145	3.606736	523.371010	62.49	46	5,460,285	323,991	*P. guariconensis*	5,091	79	52	SRS7648291	JADLJO000000000
20-MO00623	2018	Anchovy	Kumasi	146	3.571756	523.322260	63.30	169	5,288,546	71,986	*P. guariconensis*	4,710	74	46	SRS7648294	JADLJP000000000
20-MO00624	2018	Tilapia	Tamale	146	3.362602	493.079541	55.40	57	5,387,473	305,993	*P. zeshuii*	5,041	67	93	SRS7648296	JADMCD000000000
20-MO00625	2018	Bigeye grunt	Accra	146	4.897526	717.096751	63.32	121	5,186,595	145,104	*P. monteilii*	4,741	74	39	SRS7648295	JADLSK000000000
20-MO00626	2018	African moonfish	Accra	146	4.231398	620.321845	62.42	83	5,501,680	172,172	*P. monteilii*	5,098	78	69	SRS7648297	JADLJQ000000000
20-MO00627	2018	Anchovy	Accra	146	3.751486	549.815338	62.74	137	5,882,527	93,066	*P. putida*	5,357	82	52	SRS7648299	JADLJR000000000
20-MO00628	2018	Anchovy	Accra	146	4.076876	597.221091	62.83	131	5,882,156	110,679	*P. putida*	5,356	82	52	SRS7648298	JADLJS000000000
20-MO00629	2018	African moonfish	Accra	146	4.219840	619.454723	61.58	101	4,793,766	119,685	*P. putida*	4,355	79	46	SRS7648301	JADLJT000000000
20-MO00630	2018	WA pygmy herring	Bolgatanga	146	2.832042	415.657437	61.60	99	4,710,419	191,328	*P. putida*	4,374	78	46	SRS7648302	JADLJU000000000
20-MO00631	2018	WA pygmy herring	Bolgatanga	146	4.305644	631.292956	61.61	94	4,710,252	186,030	*P. putida*	4,375	78	44	SRS7648303	JADLJV000000000
20-MO00632	2018	Round sardinella	Tamale	146	3.363110	493.604577	61.78	96	4,868,050	213,857	*P. fulva*	4,432	79	61	SRS7648305	JADMAS000000000
20-MO00633	2018	Anchovy	Techiman	146	3.649412	535.446634	62.40	142	6,153,330	104,212	*P. putida*	5,775	83	61	SRS7648304	JADLJW000000000
20-MO00634	2018	Anchovy	Kumasi	146	4.021018	589.802612	61.52	87	4,908,887	191,143	*P. putida*	4,482	80	50	SRS7648306	JADLJX000000000
20-MO00635	2018	Anchovy	Techiman	146	4.092982	601.284084	61.29	190	5,066,612	103,772	*P. putida*	4,754	81	72	SRS7648307	JADLJY000000000
20-MO00636	2018	Anchovy	Bolgatanga	145	4.089862	596.317192	62.92	147	5,929,517	104,170	*P. putida*	5,394	86	74	SRS7648308	JADLJZ000000000
20-MO00637	2018	Anchovy	Bolgatanga	146	4.762456	698.752505	62.90	149	5,930,696	102,143	*P. putida*	5,387	86	76	SRS7648309	JADLKA000000000
20-MO00638	2018	Anchovy	Tamale	146	2.642248	387.672486	62.81	136	5,639,636	82,997	*P. asiatica*	5,171	77	67	SRS7648310	JADMAT000000000
20-MO00639	2018	Anchovy	Tamale	146	1.647074	241.574006	62.35	237	6,115,340	103,856	*P. asiatica*	5,726	82	120	SRS7648312	JADMAU000000000
20-MO00640	2018	Anchovy	Techiman	143	4.802152	690.821487	62.36	132	6,153,022	127,023	*P. putida*	5,787	81	59	SRS7648313	JADLKB000000000
20-MO00641	2018	Anchovy	Techiman	146	3.089492	452.919469	62.55	160	6,110,158	111,368	*P. putida*	5,695	83	58	SRS7648314	JADLKC000000000
20-MO00650	2018	Bigeye grunt	Techiman	146	3.610254	529.495604	61.81	49	4,583,286	211,866	*P. fulva*	4,164	69	41	SRS7725406	JADNYO000000000
20-MO00651	2018	Bigeye grunt	Techiman	146	3.641884	533.486812	63.77	59	4,505,983	296,201	*P. fulva*	4,123	68	62	SRS7725407	JADNYP000000000

a WA, West African.

b M, million.

Isolates were further subjected to cultivation in lysogeny broth (LB) for 24 h at 37°C for the preparation of genomic DNA with the PureLink genomic DNA kit (Invitrogen, Karlsruhe, Germany). For library preparation and whole-genome sequencing (WGS), the Nextera DNA Flex library prep kit with the IDT for Illumina Nextera DNA unique dual indexes set B and the NextSeq 500/550 midoutput kit v2.5 (300 cycles) for paired-end sequence determination (2 × 151-bp), respectively, were used on a NextSeq 500 device, as recommended by the manufacturer (Illumina, Inc., San Diego, CA, USA). The raw reads were trimmed using fastp v0.19.5 (https://github.com/opengene/fastp; parameters: base limit, 50; required length, 15) and checked with FastQC v1.0.4 (https://www.bioinformatics.babraham.ac.uk/projects/fastqc). SPAdes *de novo* assembly and genome annotation were performed using the Pathosystems Resource Integration Center (PATRIC) release 3.6.7 (8) and the Prokaryotic Genome Annotation Pipeline (PGAP; National Center for Biotechnology Information) (9), respectively. If not otherwise indicated, default parameters were used for bioinformatics analysis.

WGS provided insight into the genetic basis of fish-associated *Pseudomonas* sp. isolates from Ghana, Africa (Table 1). In addition to a reliable assignment to a *Pseudomonas* species (WGS based), which is often challenging using mass spectrometry due to their close relationship, the genome sequence data also provide an overview of the diversity of the *Pseudomonas* species occurring within Ghanaian fish products. Here, the sequences of 12 P. putida isolates, 10 P. fulva isolates, 5 P. guariconensis isolates, 3 P. aeruginosa isolates, 2 P. monteilii isolates, 2 P. asiatica isolates, and 1 P. zeshuii isolate are announced. On the basis of the sequences’ intrinsic/acquired resistance, information on the occurrence of genes involved in biocide tolerances as well as genes involved in the potential pathogenicity of the isolates for humans, animals, and plants can be used to assess if Ghanaian fish products might pose a potential health risk to the local public.

### Data availability.

The accession numbers of the whole-genome sequences and the raw sequencing read data are given in Table 1.
